# Distinguishing Pseudoprogression From True Early Progression in Isocitrate Dehydrogenase Wild-Type Glioblastoma by Interrogating Clinical, Radiological, and Molecular Features

**DOI:** 10.3389/fonc.2021.627325

**Published:** 2021-04-20

**Authors:** Mingxiao Li, Xiaohui Ren, Gehong Dong, Jincheng Wang, Haihui Jiang, Chuanwei Yang, Xuzhe Zhao, Qinghui Zhu, Yong Cui, Kefu Yu, Song Lin

**Affiliations:** ^1^Department of Neurosurgery, National Clinical Research Center for Neurological Diseases, Beijing Tiantan Hospital, Capital Medical University, Beijing, China; ^2^Department of Pathology, Beijing Tiantan Hospital, Capital Medical University, Beijing, China; ^3^Department of Radiology, Peking University Cancer Hospital, Beijing, China; ^4^Department of Pharmacy, Beijing Tiantan Hospital, Capital Medical University, Beijing, China; ^5^Beijing Key Laboratory of Brain Tumor, Center of Brain Tumor, Institute for Brain Disorders, Beijing, China; ^6^Department of Neurosurgery, Beijing Neurosurgical Institute, Capital Medical University, Beijing, China

**Keywords:** pseudoprogression, MGMT promoter methylation, subventricular zone, IDH wild-type, glioblastoma, random forest, nomogram

## Abstract

**Background:** Pseudoprogression (PsP) mimics true early progression (TeP) in conventional imaging, which poses a diagnostic challenge in glioblastoma (GBM) patients who undergo standard concurrent chemoradiation (CCRT). This study aimed to investigate whether perioperative markers could distinguish and predict PsP from TeP in *de novo* isocitrate dehydrogenase (IDH) wild-type GBM patients.

**Methods:** New or progressive gadolinium-enhancing lesions that emerged within 12 weeks after CCRT were defined as early progression. Lesions that remained stable or spontaneously regressed were classified as PsP, otherwise persistently enlarged as TeP. Clinical, radiological, and molecular information were collected for further analysis. Patients in the early progression subgroup were divided into derivation and validation sets (7:3, according to operation date).

**Results:** Among 234 consecutive cases enrolled in this retrospective study, the incidences of PsP, TeP, and neither patterns of progression (nP) were 26.1% (61/234), 37.6% (88/234), and 36.3% (85/234), respectively. In the early progression subgroup, univariate analysis demonstrated female (*OR*: 2.161, *P* = 0.026), gross total removal (GTR) of the tumor (*OR*: 6.571, *P* < 001), located in the frontal lobe (*OR*: 2.561, *P* = 0.008), non-subventricular zone (SVZ) infringement (*OR*: 10.937, *P* < 0.001), and methylated O-6-methylguanine-DNA methyltransferase (MGMT) promoter (mMGMTp) (*OR*: 9.737, *P* < 0.001) were correlated with PsP, while GTR, non-SVZ infringement, and mMGMTp were further validated in multivariate analysis. Integrating quantitative MGMTp methylation levels from pyrosequencing, GTR, and non-SVZ infringement showed the best discriminative ability in the random forest model for derivation and validation set (AUC: 0.937, 0.911, respectively). Furthermore, a nomogram could effectively evaluate the importance of those markers in developing PsP (C-index: 0.916) and had a well-fitted calibration curve.

**Conclusion:** Integrating those clinical, radiological, and molecular features provided a novel and robust method to distinguish PsP from TeP, which was crucial for subsequent clinical decision making, clinical trial enrollment, and prognostic assessment. By in-depth interrogation of perioperative markers, clinicians could distinguish PsP from TeP independent from advanced imaging.

## Introduction

Isocitrate dehydrogenase (IDH) wild-type glioblastoma (GBM), the most common primary central nervous system (CNS) malignant tumor, carries a bleak outcome (1). Though great advancements in understanding tumor-specific histological and molecular behavior have significantly improved glioma categorization and prognosis prediction, individualized therapeutic regimens that markedly prolong survival are still insufficient. Currently, maximal safe resection following concurrent chemoradiation (CCRT) plus cycles of adjuvant temozolomide (TMZ) forms the backbone of first-line treatment modality for eligible patients, but the outcome is still far from satisfactory (2).

After initial treatment, regular and meticulous follow-up strategies, including gadolinium contrast-enhancing magnetic resonance imaging (CE-MRI) check and neurological function evaluation, are highly recommended (3). Drawbacks of this procedure do exist; however, CE-MRI fails to identify pseuodprogression (PsP) from true recurrence or progression due to contrast-enhancing lesions merely reflecting blood–brain barrier disruption and agent leakage rather than active tumor infiltration. As a subacute and transient radiographic change following CCRT typically within 12 weeks, PsP displays new or progressive contrast-enhancing lesions that mimic the features of true progression (4, 5). This is indistinguishable from conventional imaging, and this dilemma poses a profound clinical challenge for subsequent decision making and survival assessment. Advanced radiological methods could provide help to some extent, but fail to predict or conclusively ascertain the occurrence of PsP or true early progression (TeP) and place another heavy financial burden on the family (6).

Previous researches have demonstrated that the presence of PsP was an effective sign of prescribed treatment protocols and portended a favorable prognosis. Those studies also showed that PsP predominately occurred in tumors with methylated O-6-methylguanine-DNA methyltransferase (MGMT) promoter (7, 8). Conversely, treatment failure to current therapeutic strategy, including the tumor residual after surgery and resistance to chemoradiotherapy, especially for tumors invading SVZ, might be the accountable reasons for TeP. Thus, integrating those clinical characteristics might build up a reliable and robust model for predicting and distinguishing PsP from TeP. However, researches in differentiating PsP from TeP merely focused on the radiological difference in advanced imaging, such as MR spectroscopy, perfusion MRI, PET CT/MRI, etc. Limited enrolled patients, inconsistent results, and difficulties in reproducibility impeded the popularization of those advanced methods into clinical application.

In this retrospective study, we first explored whether perioperative characteristics, including clinical, radiological, and molecular information, could effectively differentiate PsP from TeP, and attempted to establish a reliable model to predict PsP.

## Materials and Methods

### Patients

A cohort of 234 consecutive adult patients from March 1, 2013 to December 31, 2019, surgically treated and pathologically defined as *de novo* supratentorial IDH wild-type GBM based on 2016 WHO classification of brain tumors were included in this retrospective study (9). All tissue sections were meticulously reviewed by three senior neuropathologists to generate a consensus diagnosis. Patients with specific expansile or circumscribed pathologies including diffuse astrocytoma, IDH mutant, pilocytic astrocytoma, pleomorphic xanthoastrocytoma, glioneuronal tumors (ganglioglioma, gangliocytoma) with anaplasia, inadequate follow-up, and comorbidity or other malignancies were excluded (Supplementary Figure 1). Clinical, radiological, and pathological information was recorded.

### Radiography and Definition of Pseudoprogression, True Early Progression, and Neither Patterns of Progression

MRI studies were performed on a 3.0 T clinical scanner (Siemens Trio Tim, Germany, or GE, USA) as previously described (10). Briefly, axial plain T1, T2, and FLAIR images, in addition to axial, sagittal, and coronary contrast-enhanced T1 images, were collected and reviewed. The tumor volumes were approximately calculated as ellipsoids (4/3π × radius_x_ × radius_y_ × radius_z_) (11, 12). The resection degree was defined according to the following equation: (preoperative tumor volume – postoperative tumor volume)/preoperative tumor volume, as gross total resection (>98%, GTR), subtotal resection (90–98%, STR), and partial resection (PR, <90%) (13). Three radiologists with over 20 years of neuroimaging experience reviewed the MRI data and defined the occurrence of progressive contrast-enhancing lesions according to RANO and modified RANO criteria (3, 14).

**Figure 1:**
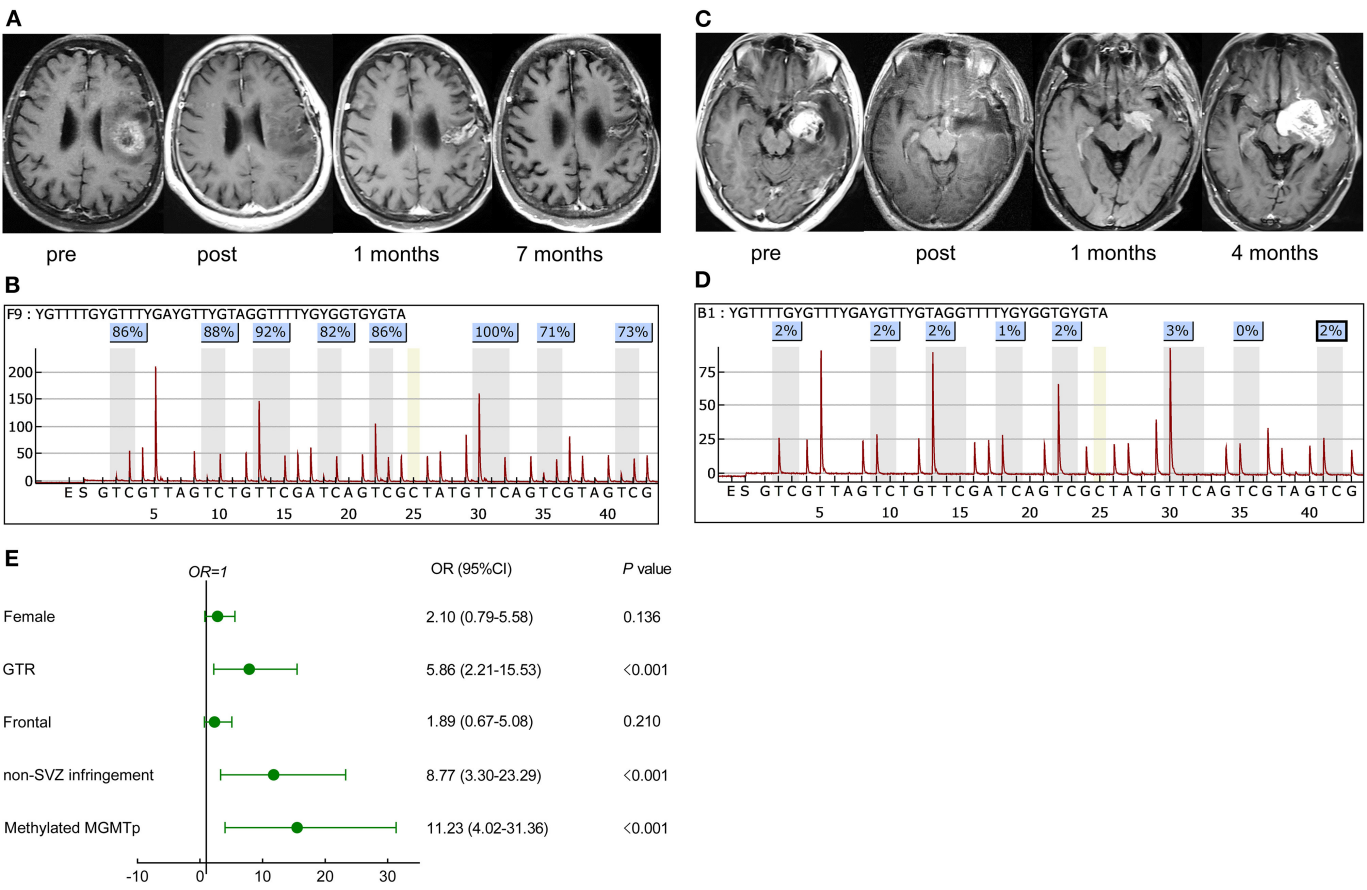
Representative cases in pseudoprogression (PsP) and true early progression (TeP) and multivariate analysis for PsP in early progression patients. **(A,C)** A 72-year-old female patient with glioblastoma, isocitrate dehydrogenase (IDH) wild-type, presented a high methylated O-6-methylguanine-DNA methyltransferase (MGMT) promoter level [average 85%, **(B)**]. The lesion that emerged 1 month after concurrent chemoradiation (CCRT) regressed spontaneously during the follow-up **(A)**. **(C,D)** A 39-year-old male patient was diagnosed with glioblastoma, IDH wild-type, and the average methylation level was 2% **(D)**. Postoperative magnetic resonance imaging (MRI) showed the tumor was totally removed. A new contrast-enhancing lesion appeared 1 month after CCRT and enlarged 3 months later, which was further pathologically confirmed as true recurrence. **(E)** Multivariate analysis demonstrated that GTR, non-SVZ infringement, methylated MGMTp were correlative factors leading to PsP. pre, preoperative MRI; post, postoperative MRI.

If patients exhibited stable disease and no evidence of lesions beyond the initial tumor boundaries based on both immediate and secondary MRI scans within 12 weeks of CCRT, they were classed as nP. In cases of lesion growth during this period (early progression), stabilized, spontaneously regressed, or even vanished lesions without further treatment were categorized as PsP (example in Figure 1A). In contrast, TeP was defined as constantly enlarging lesions and worsening neurological function deficits without discontinuation of TMZ (example in Figure 1B) (3, 4).

### Molecular Information

The 1p/19q status was determined by fluorescence *in situ* hybridization (FISH). IDH1 R132 and IDH2 R172 mutation and TERT C228T/C250T mutation were tested by Sanger sequencing. The Ki-67 index, expression of epidermal growth factor receptor (EGFR), MMP-9, TP53, vascular endothelial growth factor (VEGF), and phosphate and tension homology on chromosome 10 (PTEN) were detected by immunohistochemistry as previously described (15). The expression level of each molecule was graded as negative (–), undetermined positive (±, 0–10%), positive (+, 10–30%), median positive (++, 30–70%), and strong positive (+++, >70%). Briefly, patients were divided into high (>30%) or low (≤30%) groups for further analysis based on immunohistochemical results. BRAF V600E, FGFR1, and H3K27M mutations were evaluated by Sanger sequencing for exclusion when required.

### DNA Isolation and PSQ Testing for O-6-Methylguanine-DNA Methyltransferase Promoter Methylation

Genomic DNA was isolated from 10 formalin-fixed paraffin-embedded sections (5–8 μm) of tumor tissue with QIAamp DNA FFPE Tissue Kits (Qiagen, Germany) and further cleaned and purified. DNA concentrations were ≥30 ng/μl as assessed on a NanoDrop 2000, and ≥2 μg of sample was used for bisulfite conversion and PCR. Bisulfite-treated DNA was amplified, and eight CpGs sites containing CpGs 74–81 in exon 1 of the MGMT promoter region (genomic sequence on chromosome 10 from 131,265,507 to 131,265,544, CGctttgCGtccCGaCGccCGcaggtcctCGCGgtgCG) were tested using MGMT Methylation Detection kits (Gene Tech, China). Analyzed sequences were YGTTTTGYGTTTYGAYGTTYGTAGGTTTTYGYGGTGYGTA. Pyrosequencing was performed using a PyroMarker Q96 instrument, and data were analyzed using the PyroMarker Q96 software (Qiagen, Germany). Standards for the identification of methylated MGMTp were defined as ≥9% (16).

### Treatment and Follow-Up

All enrolled patients were surgically treated by Professor Song Lin, a neurosurgeon with experience of over 30 years. After the operation and a waiting period of about 3–5 weeks, radiation with guideline-recommended dose concurrent daily TMZ (75 mg/m^2^/day) was finished, and following cycles of maintenance TMZ (150–200 mg/m^2^ for 5 days every 28 days), adjuvant chemotherapy was administered.

CE-MRI was meticulously followed within 4 weeks after CCRT and regularly surveilled with an interval of 8–12 weeks or if necessary. PFS (progression-free survival) was defined as the duration from the initial surgery to the time of true tumor progression, which meant the time span between initial surgery and reemerged constantly enlarging lesions in the PsP group, and overall survival (OS) was termed as the duration between the initial surgery and the death, or date of the last follow-up (15, 17). Perfusion MRI by dynamic susceptibility contrast (DSC) was available to some, but not all, patients during the follow-up to provide valuable information in distinguishing PsP from TeP. Due to the nature of the retrospective study and the different machines used, we employed the mean relative cerebral blood volume (rCBV) for differentiating diagnosis (cutoff value: 1). The diagnostic sensitivity, specificity, and accuracy were assessed.

### Statistical Analysis

All statistical analyses were performed with GraphPad Prism 8.0.1 (GraphPad Software, USA), R (version 3.6.1, USA), and R studio (Version 1.2.5033, USA). The Student's *t*-test or one-way ANOVA was used for continuous variables, and the Mann–Whitney *U*-test or Kruskal–Wallis tests for non-parametric data. The Chi-square test or Fisher's exact test, as appropriate, were used to disclose associations between categorical variables. ROC curves were constructed and used to determine AUC and the optimal cutoff value by the Youden index (sensitivity + specificity – 1). Support vector machine (SVM), decision tree (DT), and random forest (RF) were applied to establish the optimal diagnostic model by ROC curves (*e1071, rpart, randomForest* packages for R). Patients in the early progression subgroup were divided into derivation (70%) and validation sets (30%) based on the date of operation in those machine learning models. The predictive performance of the nomogram was measured by the concordance index (C index), and calibration with 1,000 bootstrap samples to decrease the overfit bias (*rms* package for R). The survival rate of the patients was estimated with the Kaplan–Meier plot, and differences between curves were compared by the log-rank test. Cox proportional hazard regression model was constructed to estimate the hazard ratio (HR) for each potential prognostic factor. All tests were two sided. A *P* < 0.05 was considered statistically significant.

## Results

### Descriptive Characteristics

In this retrospective study, a total of 260 *de novo* adult supratentorial IDH wild-type GBMs were assessed, of which 26 cases were excluded due to preoperative leptomeningeal dissemination, comorbid visceral carcinoma, differential postoperative management protocols, and a loss to follow-up (Supplementary Figure 1). None of them was IDH1/2 mutant or 1p/19q co-deleted. In 161 cases with assessable TERT promoter status, 52.8% (85/161) were mutant. A total of 61 (26.1%) cases developed PsP, 88 (37.6%) suffered from TeP, and the remaining 85 (36.3%) patients were classified as nP. The differences in gender (female, 47.5 vs. 29.5%, *P* = 0.025), GTR of tumors (75.4 vs. 31.8%, *P* < 0.001), frontal location (47.5 vs. 25.0%, *P* = 0.007), non-SVZ infringement (73.8 vs. 20.5%), and mMGMTp (80.3 vs. 30.7%, *P* < 0.001, examples in Figures 1B,D) were significant between PsP and TeP subgroups (Table 1). The mean cycles of TMZ in PsP, TeP, and nP patients were also different (8.1, 6.3, 7.3, respectively, *P* = 0.009). Other clinical, radiological, and molecular characteristic comparisons were performed among the subgroups, but no disparity was observed (Table 1).

**Table 1:** Clinical, radiological, and pathological information of pseudoprogression (PsP), true early progression (TeP), neither pattern of progression (nP) patients.

**Characteristics**	**PsP**	**TeP**	***P*^**′**^-value**	**nP**	***P**″***-value**
Number of patients	61 (26.1%)	88 (37.6%)		85 (36.3%)	-
**Age at diagnosis (yrs)**					
Mean	50.4 ± 13.1	49.8 ± 12.5	0.813	48.0 ± 13.8	0.488
**Gender**					0.056
Male	32 (52.5%)	62 (70.5%)	**0.025**	58 (68.2%)	
**Preoperative KPS**					**0.002**
>70	44 (72.1%)	51 (58.0%)	0.077	70 (82.4%)	
**Tumor volume**					
Median (ml)	32.1	35.6	0.712	30.5	0.135
**Extent of resection**					** <0.0001**
GTR	46 (75.4%)	28 (31.8%)	** <0.001**	67 (78.8%)	
STR	14 (23.0%)	49 (55.7%)		17 (20.0%)	
PR	1 (1.6%)	11 (12.5%)		1 (1.2%)	
**Tumor location**					
Frontal	29 (47.5%)	22 (25%)	**0.039**	24 (28.2%)	**0.022**
Temporal	11 (18.0%)	29 (33.0%)		31 (36.5%)	
Insular	9 (14.8%)	21 (23.9%)		10 (11.8%)	
Parietal	5 (8.2%)	11 (12.5%)		8 (9.4%)	
Occipital	5 (8.2%)	1 (1.1%)		11 (12.9%)	
Others	2 (3.3%)	4 (4.5%)		1 (1.2%)	
**Non-ventricle infringement**					
Yes	45 (73.8%)	18 (20.5%)	** <0.0001**	43(50.6%)	** <0.0001**
**TERTp mutation**					
Yes	26 (55.3%)	31 (56.4%)	0.986	28(47.5%)	0.584
**MGMTp PSQ**					
Median	26%	4%	** <0.0001**	4%	** <0.0001**
Methylated	49 (80.3%)	27 (30.7%)	** <0.0001**	22(25.9%)	** <0.0001**
**Cycles of TMZ**					
Mean	8.1 (4–23)	6.3 (0–20)	**0.002**	7.3 (0–18)	**0.009**
**TP53 expression**					
High	35 (71.4%)	47 (68.1%)	0.162	41 (46.6%)	**0.027**
**MMP9 expression**					
High	14 (23.3%)	28 (32.6%)	0.226	22 (26.5%)	0.443
**EGFR expression**					
High	52 (88.1%)	74 (86.0%)	0.714	64 (77.1%)	0.153
**PTEN expression**					
High	41 (69.5%)	59 (68.6%)	0.910	50 (61.0%)	0.471
**Ki-67 level**					
High	25 (41.7%)	36 (41.9%)	0.981	34 (41.0%)	0.992

*P′-value, comparison between PsP and TeP; P″-value, comparison among groups*.

*yrs, year old; MGMTp PSQ, pyrosequencing results of MGMT promoter*.

*Bold values: significant*.

### Logistics Regression Analysis for Correlative Factors Leading to Pseudoprogression

In the early progression subgroup, univariate analysis demonstrated that female (*OR*: 2.16, 95% CI: 1.095–4.26, *P* = 0.026), GTR (*OR*: 6.57, 95% CI: 3.15–13.71, *P* < 0.001), tumor located in frontal lobe (*OR*: 2.56, 95% CI: 1.28–5.12, *P* = 0.008), non-SVZ infringement (*OR*: 10.94, 95% CI: 5.06–23.64, *P* < 0.001), and mMGMTp (*OR*: 9.74, 95% CI: 4.46–21.24, *P* < 0.001) were associated with the occurrence of PsP. Nevertheless, in the whole cohort, most GBM patients, locating in the frontal lobe were MGMT promoter methylated (53.3%, 40/75), significantly higher than non-frontal lesions (36.7%, 58/158, *P* = 0.016), but the resection degree was not statistically different (GTR: 64.0% for frontal and 58.2% for non-frontal lesions, *P* = 0.401). This result informed us that the status of MGMT was more important than the tumor location in the development of PsP. We did not observe the difference of MGMT promoter status between SVZ infringement and non-SVZ infringement patients (methylated: 36.7 vs. 48.1%, respectively, *P* = 0.079), but the resection degree was quite different (GTR: 46.1 vs. 77.4%, respectively, *P* < 0.001). Multivariate regression analysis further validated that GTR (*OR*: 5.86, 95% CI: 2.21–15.53, *P* < 0.001), non-SVZ infringement (*OR*: 8.77, 95% CI: 3.30–23.28, *P* < 0.001), and mMGMTp (*OR*: 11.23, 95% CI: 4.02–31.36, *P* < 0.001) correlated with PsP (Figure 1E), which indicated that both GTR and non-SVZ infringement were valuable features in differentiating PsP from TeP.

### The Difference in O-6-Methylguanine-DNA Methyltransferase Promoter Methylation Levels Between Pseudoprogression and Truly Early Progression Patients

**Figure 2:**
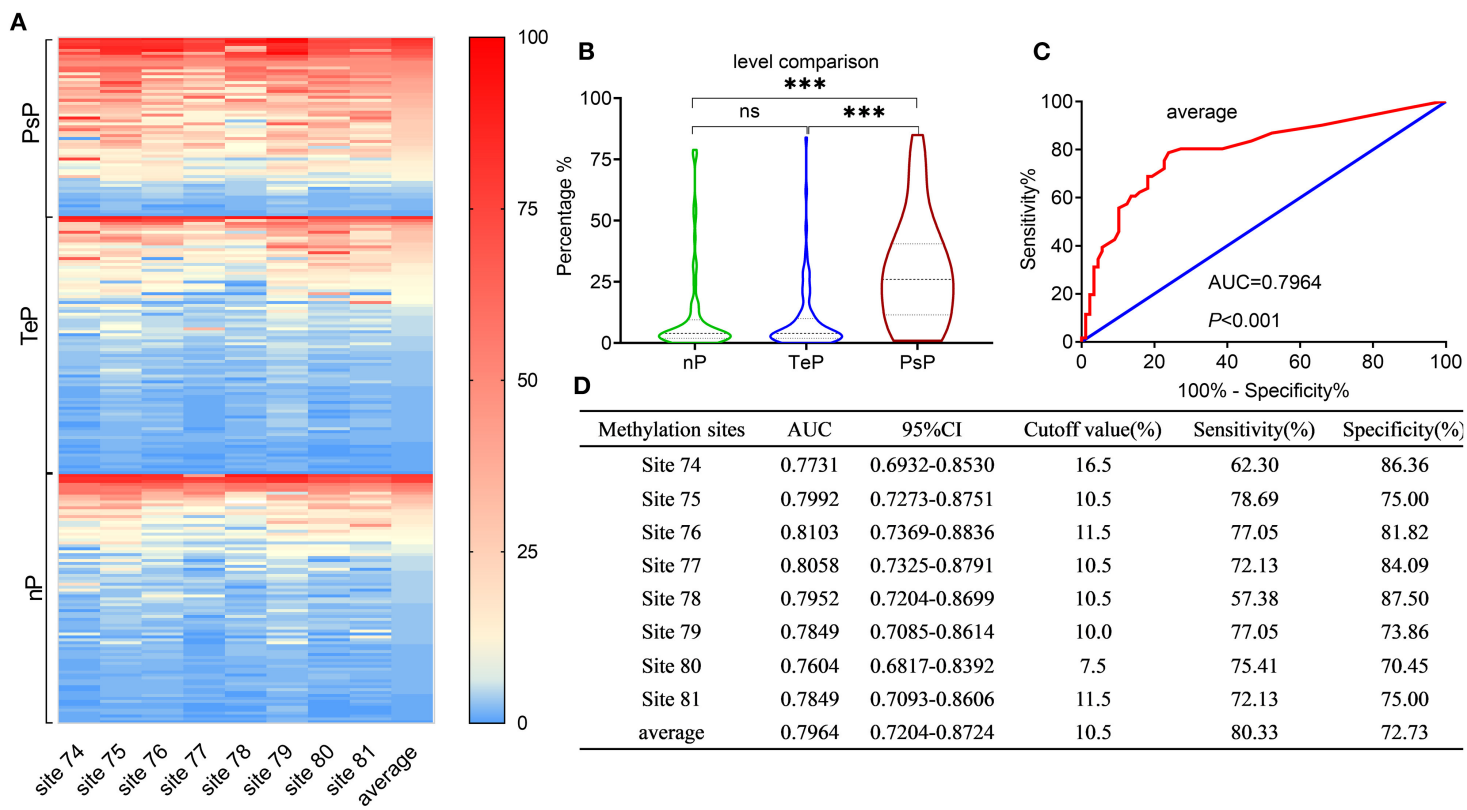
Results of MGMTp pyrosequencing (PSQ) and diagnosis accuracy for PsP. MGMTp methylation level comparison among subgroups and ROC curve for diagnosing PsP. **(A,B)** The heatmap and violin figure presented that the MGMTp methylation levels in PsP were substantially higher than those of the other groups (median 26%, 4%, 4% for PsP, TeP, and nP, respectively, Kruskal–Wallis test, *P* < 0.0001), while no difference was observed between TeP and nP (Kruskal–Wallis test, *P* = 1). The average MGMTp methylation levels showed moderate and robust differentiation efficacy compared with a single CpG site **(C,D)**. ***P < 0.0001; ns, not significant.

Binary interpretation of MGMT promoter status based on the established cutoff values of 9% might omit some useful information acquired from pyrosequencing. We next analyzed the methylation levels of the eight CpGs (74-81) in the exon 1 area of the MGMT promoter, where a substantial distinction was observed among the subgroups (median 26%, 4%, 4% for PsP, TeP, and nP, respectively, Kruskal–Wallis test, *P* < 0.0001) (Figures 2A,B).

**Figure 3:**
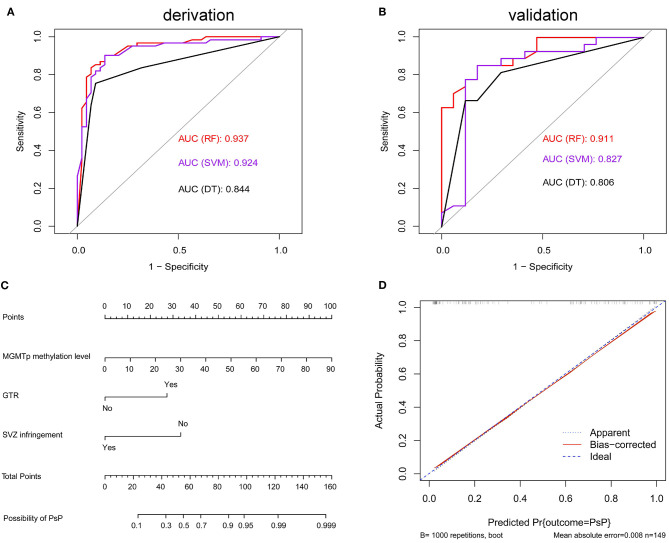
Machine learning models in differentiating PsP from TeP and nomogram in predicting PsP. **(A,B)** Random forest (RF) model showed the best discriminative ability in derivation set [*k* = 10, **(A)**] and the validation set **(B)**. **(C)** The nomogram demonstrated the relationship between perioperative markers and the possibility of developing PsP. **(D)** Calibration plots graphically showed good agreement on the presence of PsP between the possibility estimated by the nomogram.

ROC curves were used to explore the threshold identifying PsP from TeP. The average methylation levels that could diagnose PsP were 10.5% (AUC 0.7964, sensitivity 80.3%, specificity 72.7%, *P* < 0.0001) based on the maximal Youden's index (0.5483). Compared with a single methylation site, the average methylation level demonstrated the moderate discriminative performance and intermediate cutoff value (Figures 2C,D). Integrating the average MGMTp methylation levels, GTR, and non-SVZ infringement, the RF model showed the best discriminative ability compared with SVM and DT (AUC: 0.937, 0.924, 0.844 in the derivation set after 10 times of repetition, 0.911, 0.827, 0.806 in the validation set, respectively, Figures 3A,B). Detailed result comparisons are illustrated in Table 2.

**Table 2:** Model comparisons in differentiating PsP from TeP in derivation and validation set.

	**Threshold**	**Sensitivity**	**Specificity**	**PPV**	**NPV**	**Accuracy**	**Precision**	**Recall**	**F1**	**AUC**
**Derivation set**										
RF	0.253	0.836	0.932	0.944	0.804	0.876	0.944	0.836	0.887	0.937
SVM	−0.657	0.902	0.864	0.902	0.864	0.886	0.902	0.902	0.902	0.924
DT	0.604	0.754	0.909	0.920	0.727	0.819	0.92	0.754	0.829	0.8449
**Validation set**										
RF	0.629	0.852	0.824	0.885	0.778	0.841	0.885	0.852	0.868	0.911
SVM	−0.329	0.852	0.824	0.885	0.778	0.841	0.885	0.852	0.868	0.827
DT	0.902	0.667	0.882	0.900	0.625	0.750	0.900	0.667	0.766	0.806

*PPV, positive predictive value; NPV, negative predictive value; F1, F-measure (α = 1); AUC, area under the curve; RF, random forest; SVM, support vector machine; DT, decision tree*.

### Nomogram Predicted the Risk in Developing Pseudoprogression

Factors associated with PsP, including average MGMTp methylation levels, GTR, and non-VI, were used to build a nomogram to estimate the possibility of developing PsP. The resulting model was internally validated using the bootstrap validation method (B = 1,000). The nomogram showed good accuracy in estimating the possibility of PsP, and the concordance index (C-index) for the prediction nomogram was 0.916 (Figure 3C). Based on the scores acquired by each parameter, we could assess their importance in leading to the presence of PsP. Besides, calibration plots graphically showed good agreement between the actual presence of PsP and the possibility estimated by the nomogram (Figure 3D).

### Advanced Imaging Results and Pathological Findings

Additionally, we collected advanced imaging data and pathological results for PsP and TeP patients. MRI perfusion (dynamic susceptibility contrast, DSC) was available to some, but not all, patients. Among 149 patients in the TeP and PsP subgroups, 63 (42.3%) performed PWI check, including 21 PsP patients and 42 TeP patients. The sensitivity of PWI to diagnose PsP was 90.5% based on the fact that 19 PsP patients were observed with a relatively decreased blood supply (mean rCBV <1). Considering only 29 of 42 patients in the TeP subgroups showed relatively increased perfusion; the diagnostic specificity of PWI for PsP was 69.0%, and the accuracy was 76.2%. It was of note that three patients in TeP demonstrated perfusion transformation from low to high following constantly enlarged lesions, which were pathologically defined recurrence. Thus, a careful eye should be cast on the result interpretation of PWI. Pathological results were obtained from 10 TeP patients due to constantly enlarged lesions, and all were confirmed tumor recurrence. One PsP patient accepted re-operation, and only sporadic tumor cells were found. Notably, the time of re-operation was months after the initial occurrence of abnormal lesions.

### Significant Prognostic Advantage of Pseudoprogression in Progression-Free Survival and Overall Survival

**Figure 4:**
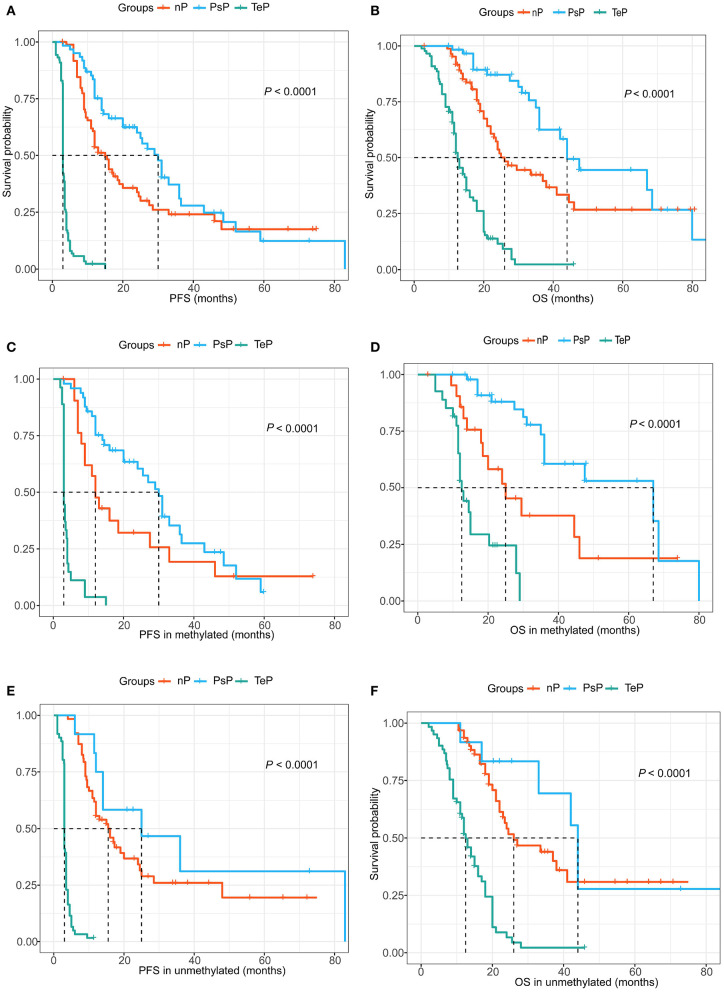
**(A,B)** PsP demonstrated favorable progression-free survival (PFS) and overall survival (OS) compared with the TeP and neither patterns of progression (nP) subgroups. **(C,D)** Extended PFS and OS were observed in the MGMTp methylated subgroup for PsP patients. **(E,F)** Similar prognostic advantage was shown for PsP patients in MGMTp unmethylated subgroup.

During data analysis, the median follow-up of the cohort was 24.0 months (range 5.0–70.0 months), and 182 (77.8%) patients suffered true progression, while 133 (56.8%) had died. In aggregate, the median PFS and OS were 9.0 and 22.0 months, respectively. Consistent with previous reports, PsP patients showed a substantially favorable prognosis compared with the TeP and nP groups. The median PFS of PsP, TeP, and nP patients were 30.0, 3.0, and 15.0 months (log-rank test, *P* < 0.0001, Figure 4A); meanwhile, the differences in OS were also significant (44.0, 12.5, and 26.0 months, respectively; log-rank test, *P* < 0.0001, Figure 4B). Furthermore, the results also demonstrated that PsP patients possessed significantly prolonged PFS and OS in MGMT promoter methylated subgroup (PFS: 30.0, 3.0, and 12.0 months; OS: 47.5, 12.5, and 30.0 months) (Figures 4C,D). Small samples in PsP (*n* = 12) from the unmethylated subgroup led to undefined survival advantage compared with nP, but still significantly exceeded TeP (PFS: 25.0, 15.5, and 3.0 months; OS: 44.0, 26.0, and 12.5 months) (Figures 4E,F). The result implicated that the superior prognosis of PsP was not totally dependent on the status of MGMTp.

**Figure 5:**
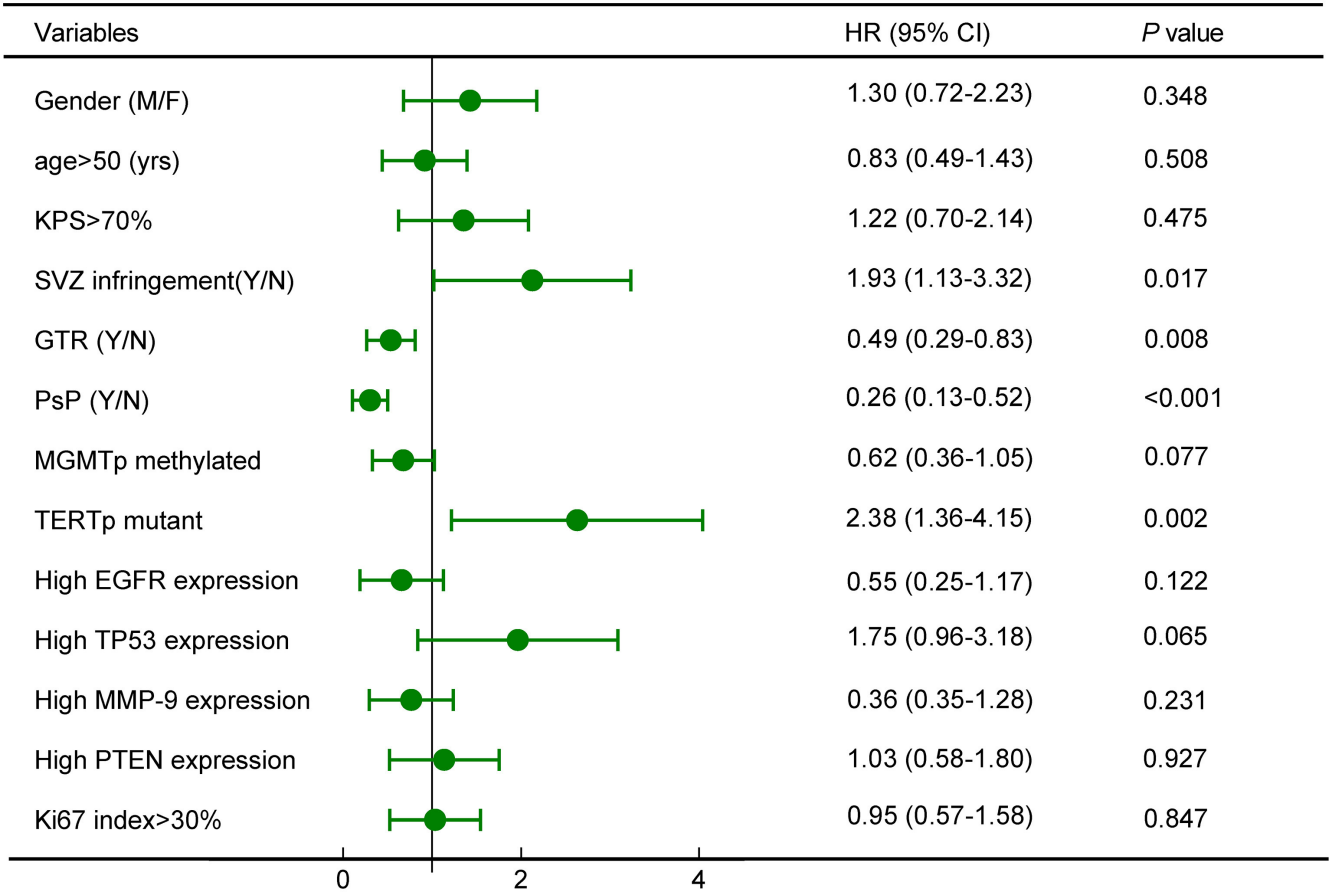
Survival analysis revealed that subventricular zone (SVZ) infringement, gross total removal (GTR), PsP, and TERTp mutation were independent prognostic factors for OS.

Cox proportional hazards model demonstrated that SVZ infringement, GTR, PsP, and TERTp mutation were independent prognostic factors for OS (all *P* < 0.05, Figure 5), while no survival differences were observed in terms of age, gender, tumor size, tumor grade, Ki67 index, expression of TP53, EGFR, MMP-9, and PTEN (Figure 5). Due to the close relationship between PsP and MGMTp status as the nomogram illustrated, we could not conclude that mMGMTp was an independent prognostic factor in multivariate analysis (*HR*: 0.62, 95% CI 0.36–1.05, *P* = 0.077, Figure 5).

## Discussion

In this retrospective study, we clarified that the combination of MGMTp methylation levels, GTR, and non-SVZ infringement could differentiate PsP from TeP with satisfying accuracy in IDH wild-type GBM. To our knowledge, this is the first study to employ quantitative MGMT promoter methylation levels and other perioperative markers to distinguish PsP from TeP, a problematic issue in clinical practice (18). It is of paramount importance to diagnose PsP and TeP for accurate prognostic evaluation, subsequent clinical decision making, and precise enrollment of true recurrent cases into clinical trials. This study provided novel insight into the diagnosis of new or progressive contrast-enhancing lesions on short-term MRI, and the results could be a robust and perfect complement to the current diagnostic strategy.

In most cases, PsP was defined retrospectively based on the improvement of subsequent MRI and clinical manifestation. PsP ranged from 20 to 30% in newly diagnosed glioblastomas, and the underlying pathophysiological mechanisms of PsP warrant further investigation (14). Notable pathological findings may include bland necrosis with prominent vascular fibrinoid necrosis, reactive gliosis, edema, demyelination, vascular hyalinization, and mixed tumor cells. Atypical histopathological changes occur in ~40% of patients who could not easily be ascribed to PsP or TeP, implying that histological reviews do not represent the “gold standard” (19). Nevertheless, identification of PsP from TeP is extremely crucial for clinical decision making because a single TMZ is justified for PsP, while intense clinical care should be recommended to TeP for potential benefits.

Advanced neuroimaging methods, such as positron emission tomography-computed tomography (PET-CT), MRI including diffusion-weighted imaging (DWI), diffusion tensor imaging (DTI), PWI, and MRS were tried to discriminate treatment-induced changes from true progression. Among them, PET CT/MRI, MRS, and PWI are the most representative and promising methods to identify PsP from TeP. PET provides penetrative insight into metabolic information for the detection of PsP, while glucose and amino acid uptake are low or absent, contrary to increased uptake in tumor progression. Studies focusing on glioblastoma progression have reported pooled sensitivity of 0.77 (95% CI: 0.66–0.85) and specificity of 0.78 (95% CI: 0.54–0.91) for ^18^F-FDG-PET (6, 20–23). Choline (Cho) reflects cell membrane turnover, which is blank in radiation necrosis. MRS employed Cho/n-acetyl-aspartate (NAA) or Cho/creatine (Cr) ratio to define recurrence with a sensitivity of 0.91 (95% CI: 0.79–0.97) and specificity of 0.95 (95% CI: 0.65–0.99) in a meta-analysis (24). The enormous difference in numerical thresholds and a strong reliance on technique and tumor type make MRS a less widespread clinical application. Dynamic susceptibility enhancement (DSC) and dynamic contrast enhancement (DCE) are representative methods to evaluate perfusion. Relative cerebral blood volume (rCBV) and relative cerebral blood flow (rCBF) reflecting tumor angiogenesis and blood supply are important parameters of PWI. In a meta-analysis, DSC perfusion had a sensitivity of 0.89 (0.83–0.94) with a specificity of 0.80 (0.72–0.86), and DCE had a sensitivity of 0.92 (0.73–0.98) with a specificity of 0.85 (0.76–0.92) for tumor progression (25). In our result, the diagnostic value of DSC was lower than reports, which might be caused by the different cutoff values and perfusion transformation from low to high in some patients. The lack of a universal threshold limits the reproducibility of those advanced imaging methods. Other drawbacks of advanced imaging lie in the disability to predict PsP, physiological uptake disturbance, and to some extent, intensifying the economic burden of patients.

Previous studies developed the concept that GBMs with methylated MGMTp were inclined to undergo PsP, while TeP predominately occurred in the unmethylated subgroup, indicating that the status of MGMTp might help to identify PsP (4, 5). The MGMT promoter is composed of 98 individual CpG sites. CpG sites 72–82 located in exon 1 predominantly control the expression of MGMT and are routinely detected for methylation status stratification (26). To date, several methods have been applied for MGMT promoter status assessments, including MSP, quantitative real-time MSP (qMSP), and methylation-sensitive high-resolution melting analysis. A drawback of MSP and qMSP is that these methods only detect a series of three to five CpG sites and fail to reflect the heterogeneity of methylation levels among the CpG sites. PSQ overcomes the heterogeneity of methylation levels at each individual CpG site and provides quantification of the MGMT promoter methylation level, which is regarded as the “gold standard” for methylated MGMT promoter confirmation (26–29). In this study, the optimal threshold for distinguishing PsP from TeP was identified. Therefore, a fresh perspective in defining specific patterns of progression was provided by perioperative markers. Though not unambiguous to rule out progression or rule in treatment-induced changes, it could be a prejudgment and complement to advanced imaging.

Park et al. reported that the EOR was an independent predictor of PsP (7). We observed this trend and found similar differences between PsP and TeP. Though the underlying mechanism why patients who underwent GTR of tumors were inclined to go through PsP remained to be elucidated, this phenomenon did do us a favor to foresee PsP to some degree. On the other hand, even tiny residuals would be the source of tumor recurrence, leading to true early progression (10, 30). The concept of gross total or even supratotal resection of GBM has been established considering the survival benefit for eligible patients (10, 31). Thus, non-GTR might play a critical role in TeP, which also implicated valuable information in differentiating PsP and TeP in turn.

Neural stem cells (NSC) with high self-renewal and proliferative capacities are predominantly located around the SVZ. GBM originating from SVZ would carry some specific characteristics of NSC, including enhanced reproductive ability, more aggressive tumor behavior, prominent resistance to radioactive therapy, etc. (31–34). Complicated mechanisms contributed to rapid tumor progression and inferior clinical outcomes in GBMs invading SVZ, including, but not limited to, tumor formation and propagation owing to aberrant Notch pathway activation and promoted invasion by pleiotrophin, which activated RhoA/ROCK signaling and weakened radiosensitivity induced by CXCL12 (stromal cell-derived factor-1), which, in turn, mediated acquired mesenchymal traits by the CXCL12/CXCR4 signaling system (35–37). On the contrary, GBM without SVZ infringement exhibited relatively gentle tumor behavior and a better response to chemoradiotherapy. Thus, as a treatment-effective sign, PsP might predominantly occur in GBMs away from SVZ.

Enormous efforts have been put into exploring therapeutic regimes that would bring survival benefit to TeP patients, but nearly none showed authentic effectiveness. Although some results were inspiring and encouraging regarding positively extended progression-free survival, for instance, bevacizumab, the vascular endothelial growth factor (VEGF)-A antibody, their failure in prolonging OS overtly disappointed neuro-oncology physicians (38). Currently, a brand-new treatment modality, electric tumor treating fields (TTF) with demonstrable antiproliferative properties, has remarkably prolonged the OS and improved the quality of life for newly diagnosed and recurrent GBM patients regardless of their molecular subtypes (39). Results of clinical trials were indeed illuminating. Recommendations of TTF to eligible patients could help to acquire survival benefits and provide an alternative or complementary treatment choice for GBM patients, especially for the TeP subgroup.

This was a retrospective study in a single neurosurgical institute and had some limitations. Analytic bias may exist due to the small sample size and the nature of the retrospective study. Molecular markers were not detected in some patients due to limited tissue samples, and other methylation testing strategies, including fewer or more CpG sites, were not employed for MGMT promoter detection. Further studies might find out more valuable markers that could diagnose PsP precisely.

## Conclusion

The combination of clinical, radiological, and molecular characteristics provided a robust method to distinguish PsP from TeP, which was of paramount significance for subsequent clinical decision making, survival prediction, and protentional clinical trial enrollment. GBM patients who presented new or progressive lesions soon after CCRT in MGMTp unmethylated, SVZ infringement, and non-GTR subgroup were prone to carry an abysmal prognosis and should be considered for a more vigorous second-line treatment modality.

## Data Availability Statement

The raw data supporting the conclusions of this article will be made available by the authors, without undue reservation.

## Ethics Statement

The studies involving human participants were reviewed and approved by Capital Medical University. The patients/participants provided their written informed consent to participate in this study.

## Author Contributions

ML, XR, HJ, CY, XZ, QZ, YC, and SL conducted the literature search, designed the study, collected and interpreted the data, followed-up the patients, and wrote the manuscript. GD, JW, and KY analyzed the data. All authors contributed to the article and approved the submitted version.

## Conflict of Interest

The authors declare that the research was conducted in the absence of any commercial or financial relationships that could be construed as a potential conflict of interest.

## Supplementary Material

Supplementary Figure 1 Patients inclusion and exclusion criteria in this study.

## Abbreviations

MGMT: O-6-methylguanine-DNA methyltransferase
PsP: pseudoprogression
TeP: true early progression
IDH: isocitrate dehydrogenase
GBM: glioblastoma
PSQ: pyrosequencing
PFS: progression-free survival
OS: overall survival.
