# Corrigendum: Distinguishing Pseudoprogression From True Early Progression in Isocitrate Dehydrogenase Wild-Type Glioblastoma by Interrogating Clinical, Radiological and Molecular Features

**DOI:** 10.3389/fonc.2021.700599

**Published:** 2021-05-19

**Authors:** Mingxiao Li, Xiaohui Ren, Gehong Dong, Jincheng Wang, Haihui Jiang, Chuanwei Yang, Xuzhe Zhao, Qinghui Zhu, Yong Cui, Kefu Yu, Song Lin

**Affiliations:** ^1^ Department of Neurosurgery, National Clinical Research Center for Neurological Diseases, Beijing Tiantan Hospital, Capital Medical University, Beijing, China; ^2^ Department of Pathology, Beijing Tiantan Hospital, Capital Medical University, Beijing, China; ^3^ Department of Radiology, Peking University Cancer Hospital, Beijing, China; ^4^ Department of Pharmacy, Beijing Tiantan Hospital, Capital Medical University, Beijing, China; ^5^ Beijing Key Laboratory of Brain Tumor, Center of Brain Tumor, Institute for Brain Disorders, Beijing, China; ^6^ Department of Neurosurgery, Beijing Neurosurgical Institute, Capital Medical University, Beijing, China

**Keywords:** pseudoprogression, MGMT promoter methylation, subventricular zone, IDH wild-type, glioblastoma, random forest, nomogram

In the published article, there was an error regarding the affiliation for Song Lin. As well as having affiliations 1 and 5, he should also have “^6^Department of Neurosurgery, Beijing Neurosurgical Institute, Capital Medical University, Beijing, China”.

The authors apologize for this error and state that this does not change the scientific conclusions of the article in any way. The original article has been updated.

